# Commentary: The distress of psychological adaptation in nutritional management among people after esophagectomy: an interpretative phenomenological study

**DOI:** 10.3389/fnut.2026.1817612

**Published:** 2026-04-15

**Authors:** Bengu Depboylu

**Affiliations:** Department of Radiation Oncology, Aydin Adnan Menderes University, Aydin, Türkiye

**Keywords:** esophageal cancer, esophagectomy, nutrition impact symptoms, nutritional management, psychological adaptation, supportive care, survivorship

## 1 Introduction

Liu et al.'s (1) interpretative phenomenological study assesses how patients with esophageal cancer manage nutrition following esophagectomy, showing that the challenges extend beyond food intake. Using semi-structured interviews with 16 participants and Interpretative Phenomenological Analysis (IPA) (2), the authors highlight that long-term nutritional impact symptoms (NISs) and post-esophagectomy eating difficulties may persist past recovery. These issues influence self-perception, family relationships, and social eating, making nutritional management a psychological aspect of survivorship rather than a practical task (3, 4).

This is a significant contribution because nutritional problems after esophagectomy are often discussed in functional or medical terms; however, the study links them to distress, role disruption, and uncertainty. That perspective matters in multidisciplinary oncology care (5).

## 2 What this paper contributes to survivorship care

Liu et al. (1) identify three cardinal themes in their IPA analysis: cognitive and behavioral adaptation to gastrointestinal symptoms, adaptive challenges and identity reconstruction within family dietary contexts, survival significance, and social pressures in nutritional management. These themes suggest that eating after esophagectomy is not simply a question of compliance with dietary advice. It becomes a site where symptoms, fear, family expectations, and altered identity converge.

Two points stand out: first, the study highlights symptom-related cognitive anxiety. When expectations are not met after discharge, patients may become overly alert and focus their life planning on reflux or dumping (1). This explains why distress may continue despite expected medical recovery.

Second, the family environment actively shapes how nutritional difficulties are experienced and managed. Liu et al. demonstrate how family support can sometimes turn into “surveillance” or “pressure,” as the family's wish for the patient to eat more conflicts with the patient's actual physical abilities, intensifying identity strain and emotional distress (6, 7).

## 3 Where the clinical translation can be enhanced

Although the study focuses on the 3–12 months post-surgery period, its clinical implications extend beyond that interval. The sense of having one's expectations violated after treatment is not a trivial adjustment problem. If unaddressed, it may harden into chronic vigilance, fear-based restriction, and isolation from social life (3, 4). The findings help identify when nutritional follow-up alone is not enough.

### 3.1 From themes to triage

The multidisciplinary aspect of postoperative nutritional care may include dietitians, the surgical/oncology team, nurses, and physical therapists, especially when recovery is affected by fatigue, deconditioning, or limited mobility. Speech-language pathologists may help with prominent dysphagia. Occupational therapists support meal preparation and adaptive self-care strategies. Psycho-oncology/psychology professionals are involved when anxiety, avoidance, or identity strain persists (8, 9).

Meal preparation can reflect autonomy; survivors who cook may increase their confidence through portioning, modifying textures, and symptom-based food choices (10). Because cooking roles are gender- and culture-dependent, stepped-care planning should assess household meal preparers and tailor support according to established distress-management guidance (11, 12).

### 3.2 Anchoring treatment context and symptom drivers

Post-esophagectomy nutritional experiences are heterogeneous and should not be interpreted in isolation from the treatment context. Symptom burden may vary according to the surgical technique, anastomotic complications, reflux, dumping syndrome, dysmotility, dysphagia, and the broader sequence of multimodal treatment (3, 13, 14). This point is especially relevant for radiation oncology teams. Swallowing difficulty, food avoidance, and altered eating behavior may reflect not only surgery itself, but also the cumulative burden of radiotherapy-related toxicity, systemic treatment, or prolonged recovery after multimodal care (5). Nutritional distress should therefore be interpreted within the patient's full treatment trajectory.

### 3.3 Family-system dynamics as an intervention target

The family dimension may be the most actionable part of the study (1). What relatives describe as “support” may be experienced by patients as pressure, correction, or a loss of control (1). One practical implication is that nutritional counseling should sometimes be delivered at the family level rather than as individual instruction alone. Expectations about portion size, pacing, and symptom limits should be discussed openly. Brief caregiver guidance may prevent support from becoming pressure (6, 7). This issue is central. Some patients experience eating distress equally from relational factors and their symptoms.

### 3.4 Rebuilding a “survivor identity” without minimizing risk

Liu et al. also highlight a dimension that is easy to miss in routine follow-up: how patients understand themselves after treatment. Nutritional recovery is not only about restoring intake, but also rebuilding competence, safety, and social belonging after a period in which eating became difficult and medically challenging (1). Survivorship care supports that process through the normalization of symptom trajectories, reinforcing skills-based self-management, and helping patients re-enter social eating gradually rather than abandoning it altogether (15–17). The aim is not to deny fear of recurrence, but to prevent fear, shame, or repeated bodily disappointment from defining everyday life more than necessary.

## 4 Discussion

Liu et al. offer a clinically useful account of why nutritional management after esophagectomy may remain distressing long after hospital discharge. A major strength of the study is that it links symptom burden with cognitive anxiety, family dynamics, and altered self-perception instead of treating eating difficulty as a simple adherence problem (1, 4). The framing fits well with wider evidence showing that NISs and nutrition remain common after esophagectomy, and are associated with worse quality of life in survivorship (3, 18).

Cancer nutrition guidelines emphasize early identification of malnutrition risk and individualized counseling, while psychosocial distress guidelines support routine screening and stepped-care management (19–21). Consequently, these frameworks suggest that post-esophagectomy care should look beyond only weight loss or caloric adequacy. Persistent NISs, fear-driven restriction, conflict around meals, and ongoing helplessness may all signal the need for broader supportive care (19, 20).

This commentary is based on a single qualitative study, and such findings are inevitably influenced by contextual factors. Their transferability is enhanced when relevant clinical details are clearly specified, including time since surgery, major complications, and concurrent therapies (22). The themes described by Liu et al. will be recognizable to clinicians involved in survivorship care and align with broader literature on symptom-driven hypervigilance, distress, and recurrence fear (15).
